# Relative biological effectiveness of simulated solar particle event proton radiation to induce acute hematological change in the porcine model

**DOI:** 10.1093/jrr/rrt108

**Published:** 2013-09-11

**Authors:** Jenine K. Sanzari, Steven X. Wan, Eric S. Diffenderfer, Keith A. Cengel, Ann R. Kennedy

**Affiliations:** Department of Radiation Oncology, Perelman School of Medicine, University of Pennsylvania, 195 John Morgan Building, 3620 Hamilton Walk, Philadelphia, PA 19104-6072, USA

**Keywords:** solar particle event, protons, blood cells, relative biological effectiveness

## Abstract

The present study was undertaken to determine relative biological effectiveness (RBE) values for simulated solar particle event (SPE) radiation on peripheral blood cells using Yucatan minipigs and electron-simulated SPE as the reference radiation. The results demonstrated a generally downward trend in the RBE values with increasing doses of simulated SPE radiation for leukocytes in the irradiated animals. The fitted RBE values for white blood cells (WBCs), lymphocytes, neutrophils, monocytes and eosinophils were above 1.0 in all three radiation dose groups at all time-points evaluated, and the lower limits of the 95% confidence intervals were > 1.0 in the majority of the dose groups at different time-points, which together suggest that proton-simulated SPE radiation is more effective than electron-simulated SPE radiation in reducing the number of peripheral WBCs, lymphocytes, neutrophils, monocytes and eosinophils, especially at the low end of the 5–10 Gy dose range evaluated. Other than the RBE values, the responses of leukocytes to electron-simulated SPE radiation and proton-simulated SPE radiation exposure are highly similar with respect to the time-course, the most radiosensitive cell type (the lymphocytes), and the shape of the dose–response curves, which is generally log-linear. These findings provide additional evidence that electron-simulated SPE radiation is an appropriate reference radiation for determination of RBE values for the simulated SPE radiations, and the RBE estimations using electron-simulated SPE radiation as the reference radiation are not complicated by other characteristics of the leukocyte response to radiation exposure.

## INTRODUCTION

On space missions outside the Earth's magnetosphere, astronauts are at risk of increased radiation exposures during a solar particle event (SPE). SPEs originate from magnetically disturbed regions of the Sun, which sporadically emit bursts of energetic charged particles [1, 2], which are predominately protons [3]. Radiation exposure in a major SPE may result in acute radiation sickness, skin injury, and/or compromised immune defense in spacecraft crew-members. Based on the historical data for the Carrington flare of 1859, the worst-case scenario estimated doses of SPE radiation can be life threatening unless substantial shielding is provided [4]. More recent SPEs (August 1972, October 1989 and September 1989) were estimated to be capable of delivering a skin dose of 32.15 Gy, 25.99 Gy and 7.68 Gy, respectively [5]. While avoidance is the best protective strategy against SPE radiation, it is nearly impossible to avoid the radiation risk completely for astronauts, since the timing and energy spectrum of SPEs cannot be predicted with acceptable accuracy. The shielding of the spacecraft and spacesuit provide modest protection [6], which is diminished during extravehicular activity (EVA). Thus, it is important to evaluate the biological effects of SPE radiation to gather critical information for risk assessment and mission planning.

SPE radiation consists mainly of protons with energies ≤ 50 MeV; however, some of the SPE protons have considerably higher energies [7]. Proton radiation with such a unique energy profile has limited penetrating ability in tissue. Thus, exposure to SPE radiation results in an inhomogeneous whole-body dose distribution, with much higher doses delivered to the skin and subcutaneous tissues than to internal organs. For astronauts engaged in an EVA, absorbed radiation doses (based on the August 1972 SPE) are estimated to be as high as 32 Gy to the skin and 1.4 Gy in the blood-forming organs. Inside the spacecraft, the doses are estimated to be ∼ 3 Gy to the skin and 0.5 Gy to the blood-forming organs [5]. The dose–toxicity relationship for SPE radiation is not well understood because conventional radiation, such as X-rays and γ-rays, which results in a homogeneous whole-body dose distribution, is only of limited value as the reference radiation to determine the relative biological effectiveness (RBE) values for SPE radiation. For example, it has been shown that the damaging effects of simulated SPE radiation on leukocytes and platelets are generally less pronounced compared with those caused by conventional radiation, and the results obtained with γ-rays, X-rays and monoenergetic protons may not necessarily predict biological responses to SPE radiation [8]. It has been further demonstrated that extrapolating proton fluence measurements from low-energy protons (≤30 MeV) to protons with energy of 100 MeV may underestimate the dose or dose equivalence by factors as large as 2–3 [9]. To provide appropriate reference radiations that can be used to determine RBE values for SPE radiation, a novel approach using megavoltage electron radiation to simulate the whole-body dose distribution of SPE radiation was developed [10]. Yucatan minipigs were chosen as the animal model for the SPE radiation dose distribution simulation because the pig model is believed to be the best model of human skin, due to the similarities in the structure and thickness of the skin layers [11–13]. In addition, pigs have also been shown to respond in a similar fashion to radiation exposure as observed for human subjects [11].

We have previously described the use of 6 + 12 MeV electron radiation to simulate proton radiation exposure from an SPE [10], and we have previously evaluated the effects of simulated proton SPE radiation on peripheral blood cell counts using the Yucatan minipig model [14]. In the present study, we determined the effects of megavoltage electron radiation on the peripheral blood cell counts using the same Yucatan minipig model and calculated RBE values for simulated proton SPE radiation. The megavoltage electron beams are composed of ∼ 80% 6-MeV electrons and 20% 12-MeV electrons, which closely matches the September 1989 SPE radiation dose distribution [10]. The RBE values and a trend established in the present study for simulated SPE radiation will be valuable for assessing the risk of hematopoietic cell damage in astronauts exposed to SPE radiation. From this point forward, simulated proton SPE radiation will be referred to as pSPE and simulated electron SPE radiation will be referred to as eSPE.

## MATERIALS AND METHODS

### Animals

Yucatan minipigs aged 8–14 weeks were purchased from Sinclair Bio Resources, LLC (Auxvasse, MO) and acclimated for 7 d in the Loma Linda University Medical Center (LLUMC) animal facility prior to beginning the experiments. The animals were housed individually with *ad lib* access to water and fed twice daily with standard minipiglet chow. The animal care and treatment procedures were approved by the Institutional Animal Care and Use Committee of the LLUMC and the University of Pennsylvania.

Upon acclimation, the animals used for the proton experiment were randomly assigned to four groups with three animals per group and exposed to pSPE radiation at doses of 0 (sham-irradiation control), 5.0, 7.7 and 10.0 Gy, as described previously [14]. In addition to the proton experiment, two separate experiments were performed with eSPE radiation at the University of Pennsylvania (PENN). In these experiments, electrons were used as the reference radiation to determine RBE values for the pSPE radiation. In the first experiment, the animals were randomly assigned into four groups with three animals per group and exposed to eSPE at doses of 0 (sham-irradiation control), 5, 7.7 and 15 Gy. In the second experiment, the animals were randomly assigned into four groups with three animals per group and exposed to eSPE at doses of 0, 7.5, 10 and 20 Gy. The animals were evaluated daily for at least 30 d after irradiation.

### Irradiation

In the pSPE radiation experiment, the animals were exposed to beams comprised of protons with energy distribution up to 155 MeV and a custom depth dose profile designed to closely resemble the September 1989 SPE radiation, as with the eSPE. The source, modulation and characterization of the proton beams as well as the proton dose calibration, delivery and monitoring have previously described in detail [14], and these details are not repeated here. Briefly, the characterization of the proton beam was completed using radiographic film, radiochromic film and ionization chambers. The Geant4-based Monte Carlo dose-modeling software tool was utilized to estimate dose to specific organs. A detailed description of the radiation transport simulation and dose distribution analysis will appear in a future publication and has been omitted here because it is beyond the scope of this work. Briefly, a model of the electron beam used in these studies was developed and validated against measurements of lateral and depth dose profiles of the electron beam used at PENN. The proton beam was modeled as a broad parallel beam with energies chosen to closely match the depth dose profile of the proton beam provided at LLUMC. The software incorporates computed tomography (CT) images of appropriately sized Yucatan minipigs to construct the computational geometry which was used for the calculation of 3-D dose distributions during the radiation transport simulation. The experiments reported here were simulated using the electron and proton beam models with beam angles varied to correspond to the average animal orientation during irradiations at PENN and LLUMC. Organ doses were computed for each irradiation scenario by accumulating the 3-D dose distribution within organ volumes, which were delineated on the same CT sets used in these simulations. For both the electron and proton irradiation exposures, animals were not anesthetized but restrained in custom-made, aerated plexiglass chambers ∼ 31 (w) × 69 (l) × 36 cm (h). Animal exposures were ∼ 3 h long, with a dose rate of 0.83 Gy/min for the pSPE exposures and an average dose rate of 1.1 Gy/min for the eSPE exposures. To ensure uniform whole-body irradiation, irradiation chambers were rotated at regular dose intervals.

Irradiations performed with protons and this energy/fluence profile are referred to in this manuscript as pSPE. For the eSPE radiation experiments, the animals were irradiated with eSPE beams composed of a mixture of 80% 6-MeV electrons and 20% 12-MeV electrons, which closely mimics the whole-body dose distribution of the 1989 SPE [10]. Irradiations performed with electrons and this energy/fluence profile are referred to as eSPE.

### Blood cell count analyses

At 4 h, at 1, 4 (protons only), 7 (electrons only), 14 and 30 d after the proton or electron radiation exposure, a whole-blood sample was collected from each animal (cranial vena cava), and placed into a collection tube containing EDTA. The blood samples (refrigerated) were sent to Antech Diagnostics (Irvine, CA, with numerous laboratories in the USA) and analyzed using a Bayer Advia 120 Hematology Analyzer within 24 h of the blood sample collection.

### Data and statistical analyses

The mean counts of WBCs, lymphocytes, neutrophils, monocytes, eosinophils, red blood cells (RBCs) and platelets of all animals before irradiation were calculated for each experiment and used as the baseline control values for the respective blood cell types in the same experiment. For each animal at each time-point after irradiation, the count of each blood cell type was divided by the respective baseline control value, and the result was expressed as a fraction of the control for further analyses.

The relationship between eSPE radiation dose and the result of each blood cell type was determined for each time-point after irradiation by fitting the data to a linear quadratic model, *y* = e^−a*D*−b*D*2^, in which *D* is the radiation dose and *y* is the blood cell count expressed as a fraction of control.

To determine the RBE, which is defined as the ratio of the dose of a reference radiation that produces a particular effect or outcome to the dose of the investigational radiation (the pSPE in this case) for this same effect, the dose of eSPE radiation required to produce the same hematological effect observed in each proton-irradiated animal was estimated from the non-linear regression line. The ratio of eSPE dose to proton radiation dose producing the same hematological effect was calculated. The RBE values for individual animals were plotted against proton radiation doses to illustrate the relationship between the RBE value and the proton radiation dose. Since the blood cell count was performed on Day 7 but not on Day 4 after eSPE irradiation, the blood cell count results obtained on Day 4 after proton irradiation were compared with the respective blood cell count results obtained on Day 7 after 6 + 12 MeV eSPE radiation for the RBE calculation.

All curve fitting and statistical analyses were performed using SigmaPlot software, version 12 (SPSS Inc., Chicago, IL) and Minitab statistical software, release 15 (Minitab, Inc., State College, PA).

## RESULTS

**Fig. 1.**
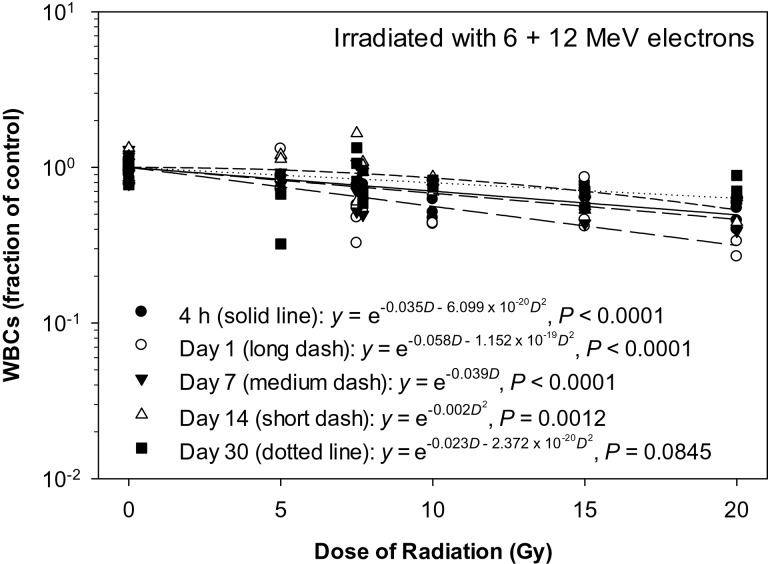
Dose response of WBCs in Yucatan minipigs after electron irradiation. The WBC data for different electron radiation dose groups at different time-points after irradiation are presented in Table 1. The dose–response curve for each time-point after irradiation is fitted using a linear quadratic model.

The present study was performed to determine the RBE values for the effects of the pSPE radiation on peripheral blood cells using Yucatan minipigs as an experimental model and eSPE radiation as the reference radiation. The peripheral blood count data at different time-points after the eSPE irradiation were fitted using a linear quadratic model to analyze the dose–response relationship for the respective blood cell types. The peripheral blood cell count data and the dose responses in the animals exposed to pSPE radiation were reported previously [14] and are not repeated here. In the animals irradiated with eSPE radiation, WBCs decreased significantly in a dose-dependent manner at 4 h and on Day 1, 7 and 14 after irradiation (Fig. 1). The linear component of the dose–response curve slope was 0.035, 0.058, 0.039 and 0.000, respectively, for 4 h, Day 1, 7 and 14 after irradiation, which were only ∼ 60% or less of the linear component of the proton dose–response curve slope for the corresponding time-points reported previously, except for the 4-h time-point when there was no significant proton dose response [14]. The steepest slope was observed on Day 1 after irradiation. The quadratic component of the dose–response curve slope was negligible (≤1.152 × 10^−^^19^) for all time-points investigated except for Day 14 after irradiation.

**Table 1.** WBC counts in animals irradiated with 6 + 12 MeV electrons

Time after irradiation	WBC counts (fraction of control) in pigs irradiated at dose shown below											
	5 Gy		7.5 Gy		7.7 Gy		10 Gy		15 Gy		20 Gy	
	Mean ± SE	Change (%)	Mean ± SE	Change (%)	Mean ± SE	Change (%)	Mean ± SE	Change (%)	Mean ± SE	Change (%)	Mean ± SE	Change (%)
Pre-irradiation	1.00 ± 0.05	N/A	1.00 ± 0.05	N/A	1.00 ± 0.05	N/A	1.00 ± 0.05	N/A	1.00 ± 0.05	N/A	1.00 ± 0.05	N/A
4 h	0.83 ± 0.05	−17.2	0.61 ± 0.09	−39.3	0.80 ± 0.10	−19.7	0.54 ± 0.04	−46.3*	0.76 ± 0.02	−23.6	0.53 ± 0.04	−46.9*
Day 1	0.96 ± 0.18	−4.1	0.45 ± 0.07	−54.9	0.59 ± 0.03	−41.5	0.44 ± 0.00	−56.2**	0.58 ± 0.14	−42.0#	0.33 ± 0.04	−66.6***
Day 7	0.85 ± 0.04	−15.0	0.76 ± 0.16	−24.0	0.60 ± 0.06	−39.6	0.77 ± 0.02	−22.7	0.56 ± 0.06	−44.3*	0.47 ± 0.07	−53.3**
Day 14	1.18 ± 0.02	17.5	1.01 ± 0.32	1.4	1.02 ± 0.04	1.5	0.79 ± 0.05	20.7	0.56 ± 0.15	44.0*	0.59 ± 0.07	−41.2#
Day 30	0.56 ± 0.12	−44.1	1.07 ± 0.15	7.0	0.75 ± 0.11	−25.4	0.78 ± 0.02	−21.8	0.65 ± 0.05	−34.8	0.74 ± 0.08	−26.2

Statistical significance for comparison with the pre-irradiation value by the Tukey test is indicated by symbols of #(*P* < 0.10), *(*P* < 0.05), **(*P* < 0.01) and ***(*P* < 0.001), respectively.

**Fig. 2.**
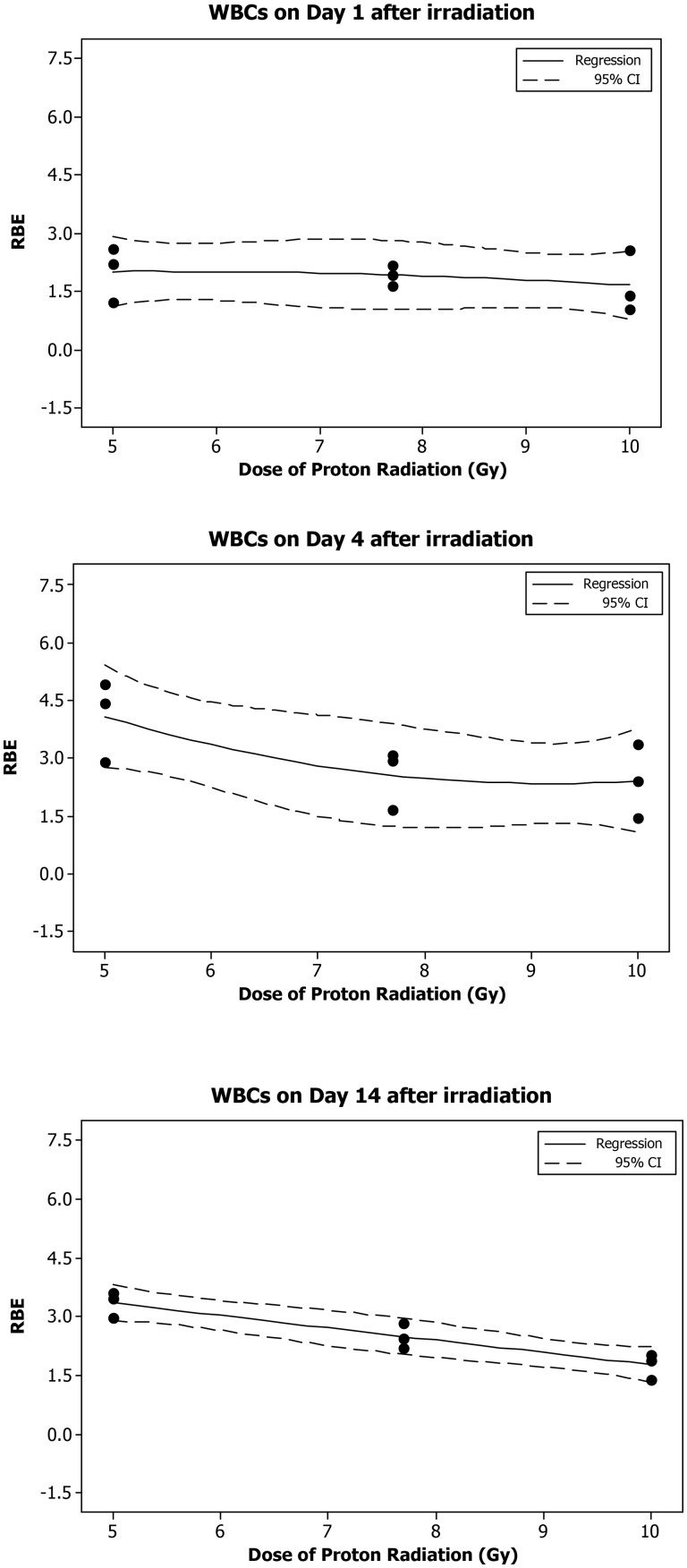
Trend of RBE values for the effects of SPE-like proton radiation on WBCs in Yucatan minipigs after irradiation. The fitted RBE values and associated 95% confidence intervals for the effect of simulated SPE radiation on WBCs are presented in Table 2. The curves are fitted using a quadratic model, and the 95% confidence interval is indicated by the dotted lines.

Based on the dose–response curve established in the animals irradiated with eSPE radiation (Fig. 1) and the WBC count data reported previously for animals exposed to pSPE radiation [14], RBE values were calculated for the effect of pSPE radiation on WBCs in irradiated animals. The results indicate that the RBE for the effect of pSPE radiation on WBCs varied with both the radiation dose and the time after irradiation (Fig. 2). The RBE displayed a slightly downward trend on Day 1 (Fig. 2A) and a clear downward trend on Day 4 (Fig. 2B) and Day 14 (Fig. 2C) after irradiation, with the increase in the pSPE radiation dose. The fitted RBE values were > 1 at all three pSPE radiation dose levels, and the lower limits of the 95% confidence interval for RBE values were > 1 for all dose levels on Day 1, 4 and 14 except for 10 Gy on Day 1. The RBE trend for WBCs was not evaluated for 4 h or Day 30 after irradiation because no significant dose response was observed at 4 h after exposure to simulated pSPE radiation [14] or on Day 30 after eSPE irradiation (Fig. 1).

**Table 2.** Relationship between RBE and proton radiation dose for WBCs

Time after irradiation	Dose of proton radiation (Gy)	RBE		
		Fitted value	95% confidence interval	
			Lower limit	Upper limit
4 h	5	0.9348	0.6139	1.2557
	7.7	**0**	−0.3209	**0.3209**
	10	**0.1904**	−0.1305	**0.5113**
1 d	5	**2.009**	**1.122**	**2.896**
	7.7	**1.921**	**1.034**	**2.808**
	10	1.661	0774	2.548
4 d	5	**4.078**	**2.745**	**5.410**
	7.7	**2.547**	**1.214**	**3.879**
	10	**2.409**	**1.077**	**3.742**
14 d	5	**3.344**	**2.886**	**3.802**
	7.7	**2.483**	**2.025**	**2.941**
	10	**1.764**	**1.306**	**2.222**
30 d	5	2.018	−1.489	5.525
	7.7	3.023	−0.484	6.530
	10	1.618	−1.889	5.125

**Fig. 3.**
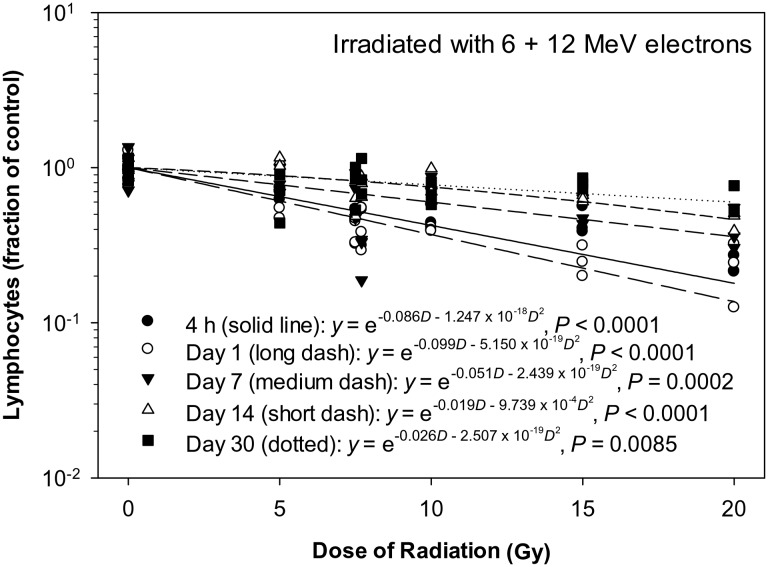
Dose response of lymphocytes in Yucatan minipigs after electron irradiation. The lymphocyte data for different electron radiation dose groups at different time-points after irradiation are presented in Table 3. The dose–response curve for each time-point after irradiation is fitted using a linear quadratic model.

**Fig. 4.**
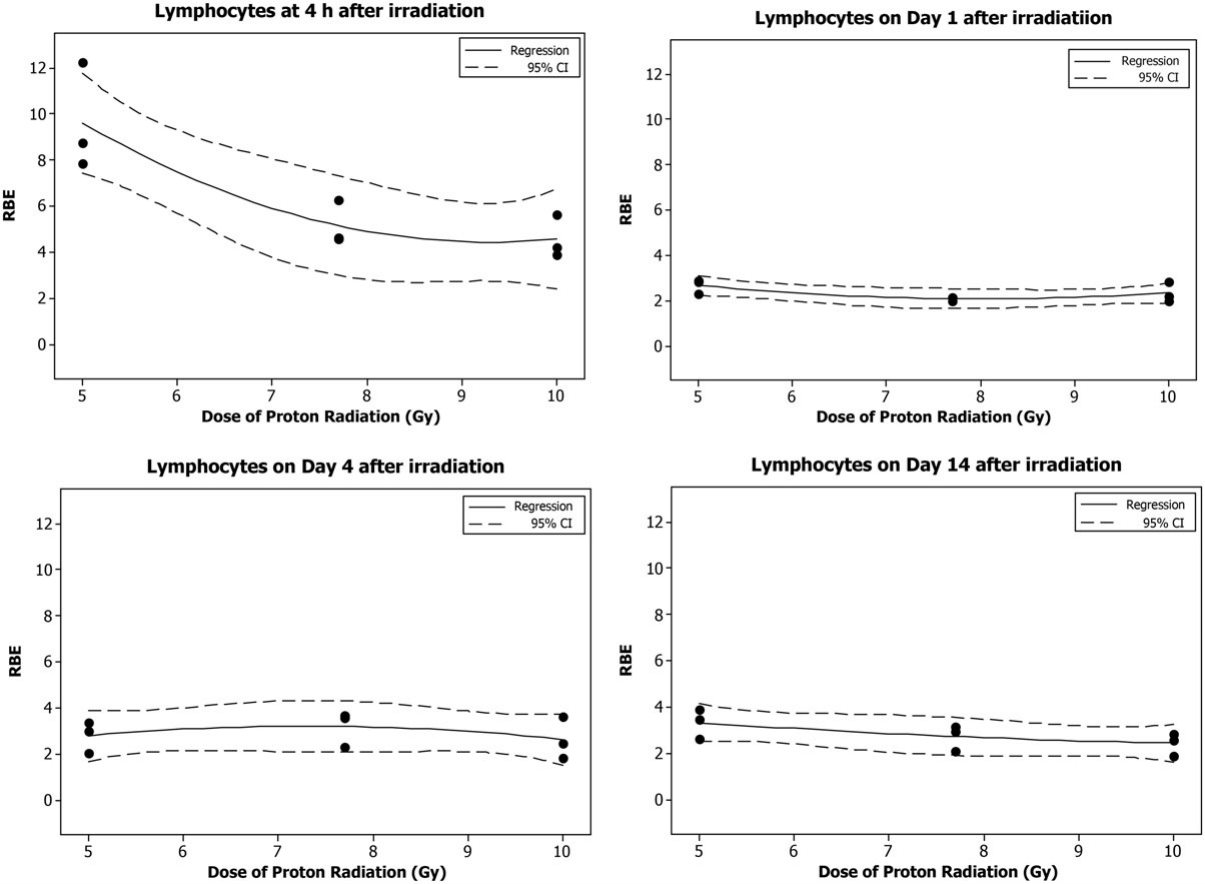
Trend of RBE values for the effects of SPE-like proton radiation on lymphocytes in Yucatan minipigs after irradiation. The fitted RBE values and associated 95% confidence intervals for the effect of simulated SPE radiation on lymphocytes are presented in Table 4. The curves are fitted using a quadratic model, and the 95% confidence interval is indicated by the dotted lines.

In the animals irradiated with eSPE radiation, the lymphocyte count decreased significantly in a dose-dependent manner at all time-points investigated (Fig. 3). The linear component of the dose–response curve slope was 0.086, 0.099, 0.051, 0.019 and 0.026, respectively, for 4 h Day 1, 7, 14 and 30 after irradiation, which was only 50% or less of the linear component of the proton dose–response curve slope for the corresponding time-points reported previously except for the Day 30 time-point, when there was no significant proton dose response [14]. The steepest slope was observed on Day 1 after irradiation. The quadratic component of the dose–response curve slope was negligible (≤1.247 × 10^−^^18^) for all time-points investigated except Day 14. Based on the dose–response curve established in the animals irradiated with eSPE radiation (Fig. 3) and the lymphocyte count data reported previously for animals exposed to pSPE radiation [14], RBE values were calculated for the effect of pSPE radiation on lymphocytes. At 4 h after irradiation, the fitted RBE value decreased from 9.60 at 5 Gy to 4.60 at 10 Gy (Fig. 4A). The trend for RBE values was relatively flat on Day 1 (Fig. 4B) and Day 4 (Fig. 4C) and slightly downward with the increase in the pSPE radiation dose on Day 14 (Fig. 4D) after irradiation. The lower limits of the 95% confidence interval for RBE values were > 1.00 for all pSPE radiation doses at all time-points up to Day 14 after irradiation. The RBE trend for lymphocytes was not evaluated for Day 30 after irradiation because no significant dose response was observed on Day 30 after exposure to pSPE radiation [14].

**Table 3.** Effective doses (and confidence intervals) of eSPE and pSPE radiation to decrease hematopoietic cells by 10% (ED_10_) and 50% (ED_50_)^a^

Time after irradiation	6 + 12 MeV electron irradiation		SPE-like proton irradiation	
	ED_10_	ED_50_	ED_10_	ED_50_
WBCs				
4 h	3.0 (2.2–3.8)	19.7 (14.4–25.0)	NM^b^	NM
1 d	1.8 (1.3–2.4)	12.0 (8.4–15.5)	**1.0 (0.8–1.3)**^**c**^	**6.7 (5.2–8.2)**
7/4 d^d^	2.7 (2.0–3.5)	18.0 (13.1–22.9)	**1.0 (0.7–1.3)**	**6.4 (4.5–8.4)**
14 d	8.2 (5.7–10.6)	>20.0	**1.3 (1.0–1.6)**	**8.3 (6.3–10.3)**
30 d	4.6 (2.2–7.0)	>20.0	2.8 (0.2–5.4)	>10.0
Lymphocytes				
4 h	1.2 (0.9–1.6)	8.1 (5.9–10.2)	**0.6 (0.4–0.8)**	**4.0 (2.6–5.5)**
1 d	1.1 (0.9–1.2)	7.0 (5.8–8.1)	**0.4 (0.3–0.6)**	**2.9 (2.0–3.9)**
7/4 d	2.1 (1.3–2.8)	13.5 (8.8–18.2)	**0.7 (0.6–0.9)**	**4.9 (3.7–6.0)**
14 d	4.5 (2.2–6.7)	18.6 (9.2–28.0)	**1.0 (0.8–1.2)**	**6.4 (5.2–7.7)**
30 d	4.1 (2.5–5.7)	>20.0	NM	NM
Neutrophils				
4 h	NM	NM	NM	NM
1 d	NM	NM	2.0 (0.8–3.1)	> 10.0
7/4 d	4.7 (2.1–7.3)	> 20.0	**1.1 (0.5–1.7)**	**7.3 (3.6–11.1)**
14 d	NM	NM	1.4 (0.8–2.2)	9.2 (5.0–13.3)
30 d	NM	NM	1.5 (0.5–2.5)	9.8 (3.0–16.5)
Monocytes				
4 h	1.2 (0.9–1.4)	7.6 (5.9–9.3)	**0.7 (0.1–1.3)**	4.8 (0.8–8.8)
1 d	1.4 (0.9–1.9)	9.3 (6.2–12.5)	1.3 (0.3–2.3)	7.3 (1.6–13.0)
7/4 d	NM	NM	NM	NM
14 d	8.6 (5.5–11.7)	>20.0	**1.6 (0.3–2.9)**	>10.0
30 d	NM	NM	NM	NM
Eosinophils				
4 h	5.4 (2.5–8.3)	13.9 (6.4–21.3)	NM	NM
1 d	2.4 (1.1–3.7)	15.8 (7.6–24.1)	**0.7 (0.0–1.5)**	**4.9 (0.2–9.7)**
7/4 d	1.3 (0.7–1.9)	8.6 (4.8–12.5)	NM	NM
14 d	4.4 (2.6–6.3)	11.4 (6.7–16.1)	**0.7 (0.4–1.1)**	**4.7 (2.5–6.9)**
30 d	NM	NM	NM	NM
Platelets
4 h	NM	NM	NM	NM
1 d	NM	NM	NM	NM
7/4 d	6.0 (3.1–9.0)	>20.0	NM	NM
14 d	2.4 (1.6–3.2)	15.8 (10.8–20.8)	4.9 (1.9–7.9)	>10.0
30 d	NM	NM	NM	NM^b^

^a^The calculated ED_90_ values were higher than the highest dose of 6 + 12 MeV electron radiation (20 Gy) or simulated SPE radiation (10 Gy) used in the experiments except for Lymphocyte count on Day 1 after the simulated SPE irradiation, for which the ED_90_ value was 9.8 (6.6–12.9). The ED_90_ values are not presented for all other treatment groups and time-points to avoid extrapolation of the dose–response curves beyond the radiation dose ranges used in the experiments. ^b^NM = not meaningful due to insignificant dose–response curve slope. ^c^The bold font indicates that the ED_10_ and/or ED_50_ values for the simulated SPE radiation were significantly different from those for the 6 + 12 MeV electron radiation. ^d^Observations were made on Day 7 after the 6 + 12 MeV electron irradiation and on Day 4 after the simulated SPE radiation.

**Table 4.** Lymphocyte counts in animals irradiated with 6 + 12 MeV electrons

Time after irradiation	Lymphocyte counts (fraction of control) in pigs irradiated at dose shown below											
	5 Gy		7.5 Gy		7.7 Gy		10 Gy		15 Gy		20 Gy	
	Mean ± SE	Change (%)	Mean ± SE	Change (%)	Mean ± SE	Change (%)	Mean ± SE	Change (%)	Mean ± SE	Change (%)	Mean ± SE	Change (%)
Pre-irradiation	1.00 ± 0.05	N/A	1.00 ± 0.05	N/A	1.00 ± 0.05	N/A	1.00 ± 0.05	N/A	1.00 ± 0.05	N/A	1.00 ± 0.05	N/A
4 h	0.44 ± 0.22	−55.7***	0.44 ± 0.06	−55.7***	0.47 ± 0.08	−53.2***	0.49 ± 0.05	−51.3***	0.45 ± 0.06	−54.7***	0.23 ± 0.02	−76.7***
Day 1	0.59 ± 0.08	−41.1*	0.42 ± 0.04	−58.4***	0.41 ± 0.08	−59.2***	0.40 ± 0.01	−60.0***	0.25 ± 0.03	−74.6***	0.23 ± 0.06	−77.0***
Day 7	0.82 ± 0.09	−17.8	0.71 ± 0.13	−28.9#	0.29 ± 0.05	−71.3***	0.76 ± 0.06	−23.9	0.51 ± 0.06	−48.7***	0.41 ± 0.07	−59.5***
Day 14	1.09 ± 0.04	9.4	0.65 ± 0.10	−35.1*	0.77 ± 0.07	−22.9	0.77 ± 0.11	−23.4	0.63 ± 0.02	−36.8**	0.46 ± 0.04	−53.7***
Day 30	0.69 ± 0.03	−31.5#	0.80 ± 0.14	−20.0	0.89 ± 0.14	−11.3	0.69 ± 0.07	−31.5*	0.79 ± 0.04	−20.6	0.61 ± 0.08	−39.2**

Statistical significance for comparison with the pre-irradiation value by the Tukey test is indicated by symbols of #(*P* < 0.10), *(*P* < 0.05), **(*P* < 0.01) and ***(*P* < 0.001), respectively.

**Fig. 5.**
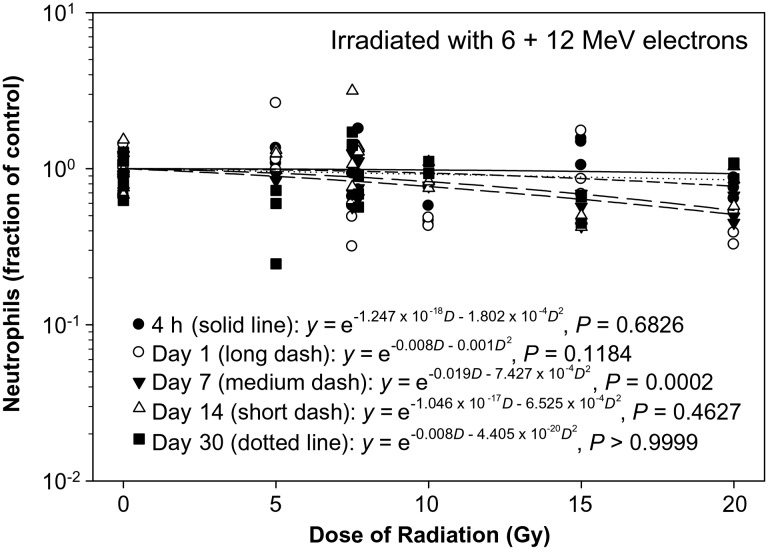
Dose–response relationship for neutrophils in Yucatan minipigs after electron irradiation. The neutrophil data for different electron radiation dose groups at different time-points after irradiation are presented in Table 5. The dose–response curve for each time-point after irradiation is fitted using a linear quadratic model.

**Fig. 6.**
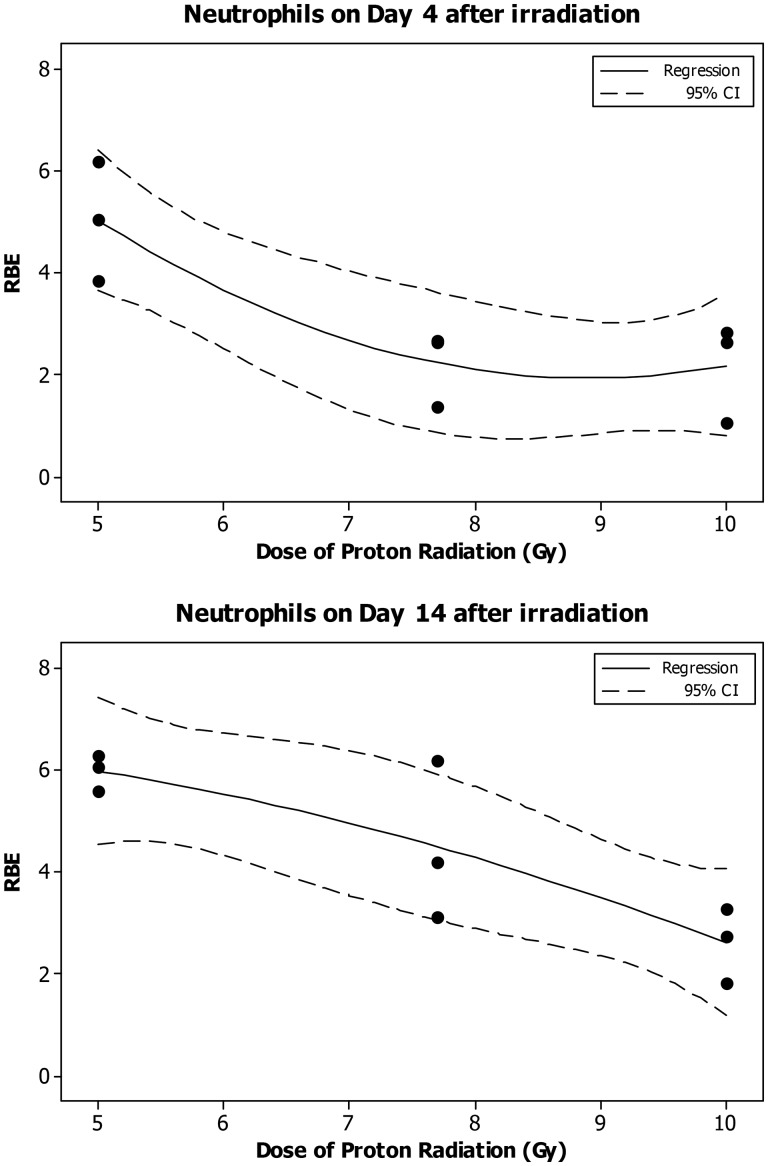
Trend of RBE values for the effects of SPE-like proton radiation on neutrophils in Yucatan minipigs after irradiation. The fitted RBE values and associated 95% confidence intervals for the effect of simulated SPE radiation on neutrophils are presented in Table 6. The curves are fitted using a quadratic model, and the 95% confidence interval is indicated by the dotted lines.

The neutrophil count decreased significantly in a dose-dependent manner only on Day 7 after the eSPE irradiation (Fig. 5). The linear component of the dose–response curve slope was only approximately 20% of the linear component of the proton dose response curve slope for the corresponding time-point reported previously [14]. The results of the dose–response curve slope on Day 14 were similar to those results obtained from the Day 7 data, although they did not reach statistical significance due to a relatively large individual variation observed at some dose levels. Based on the dose–response curve established in the animals irradiated with eSPE radiation (Fig. 5) and the neutrophil count data reported previously for each of the animals irradiated with pSPE radiation [14], RBE values were calculated for the effect of pSPE radiation on neutrophils on Day 4 (Fig. 6A) and Day 14 (Fig. 6B) after irradiation. The fitted RBE value for the effects of pSPE radiation in neutrophils displayed a noticeably downward trend. The fitted RBE values were > 1 for all three radiation dose levels and the lower limits of the 95% confidence interval for RBE values were > 1 for 5 Gy on Day 4 and all three dose levels on Day 14 after irradiation. The RBE trend for neutrophils was not evaluated for 4 h or Day 1 and Day 30 after irradiation because no significant dose response was observed at 4 h after exposure to pSPE radiation [14] or on Days 1 and 30 after the eSPE irradiation (Fig. 5).

**Table 5.** Relationship between RBE and proton radiation dose for lymphocytes

Time after irradiation	Dose of proton radiation (Gy)	RBE		
		Fitted value	95% confidence interval	
			Lower limit	Upper limit
4 h	5	**9.597**	**7.430**	**11.764**
	7.7	**5.147**	**2.981**	**7.314**
	10	**4.568**	**2.401**	**6.734**
1 d	5	**2.679**	**2.238**	**3.119**
	7.7	**2.093**	**1.653**	**2.534**
	10	**2.332**	**1.891**	**2.772**
4 d	5	**2.796**	**1.686**	**3.906**
	7.7	**3.198**	**2.088**	**4.308**
	10	**2.641**	**1.531**	**3.751**
14 d	5	**3.323**	**2.510**	**4.136**
	7.7	**2.726**	**1.913**	**3.539**
	10	**2.442**	**1.629**	**3.255**
30 d	5	1.800	−0.316	3.915
	7.7	2.192	0.076	4.307
	10	1.023	−1.093	3.138

**Table 6.** Neutrophil counts in animals irradiated with 6 + 12 MeV electrons

Time after irradiation	Neutrophil counts (fraction of control) in pigs irradiated at dose shown below											
	5 Gy		7.5 Gy		7.7 Gy		10 Gy		15 Gy		20 Gy	
	Mean ± SE	Change (%)	Mean ± SE	Change (%)	Mean ± SE	Change (%)	Mean ± SE	Change (%)	Mean ± SE	Change (%)	Mean ± SE	Change (%)
Pre-irradiation	1.00 ± 0.10	N/A	1.00 ± 0.10	N/A	1.00 ± 0.10	N/A	1.00 ± 0.10	N/A	1.00 ± 0.10	N/A	1.00 ± 0.10	N/A
4 h	1.19 ± 0.08	19.3	0.72 ± 0.11	−27.6	1.42 ± 0.19	42.4	0.59 ± 0.10	−40.7	1.37 ± 0.16	36.5	0.76 ± 0.07	−24.4
Day 1	1.59 ± 0.52	59.6	0.47 ± 0.08	−53.4	0.86 ± 0.06	−14.2	0.46 ± 0.02	−54.0	1.10 ± 0.33	9.7	0.42 ± 0.06	−58.4
Day 7	0.87 ± 0.02	−13.4	0.84 ± 0.21	−16.2	0.84 ± 0.14	−16.1	0.82 ± 0.04	−18.5	0.56 ± 0.08	−43.6	0.54 ± 0.07	−46.1
Day 14	1.27 ± 0.02	27.1	1.66 ± 0.75	66.1	1.34 ± 0.03	33.7	0.89 ± 0.11	−10.6	0.46 ± 0.02	−53.8	0.82 ± 0.13	−17.8
Day 30	0.52 ± 0.14	−47.7	1.52 ± 0.10	52.0	0.72 ± 0.10	−28.1	0.99 ± 0.06	−0.6	0.60 ± 0.07	−40.5	1.01 ± 0.07	1.0

**Fig. 7.**
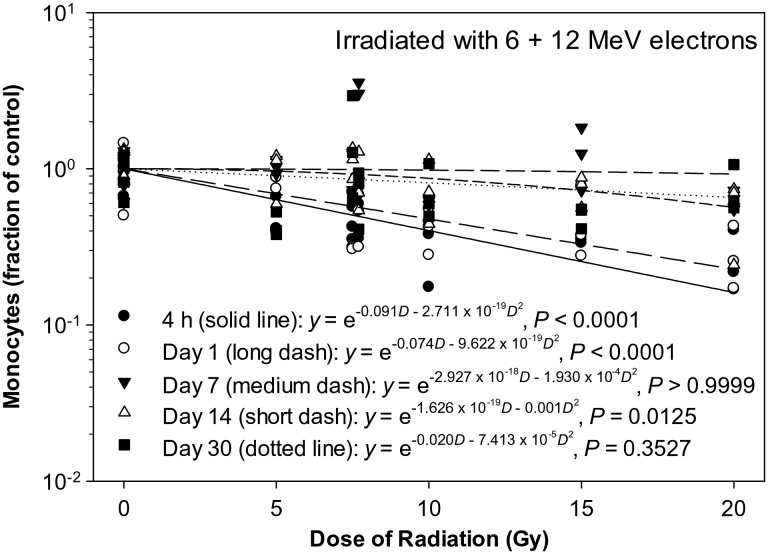
Dose response of monocytes in Yucatan minipigs after electron irradiation. The monocyte data for different electron radiation dose groups at different time-points after irradiation are presented in Table 7. The dose–response curve for each time-point after irradiation is fitted using a linear quadratic model.

**Fig. 8.**
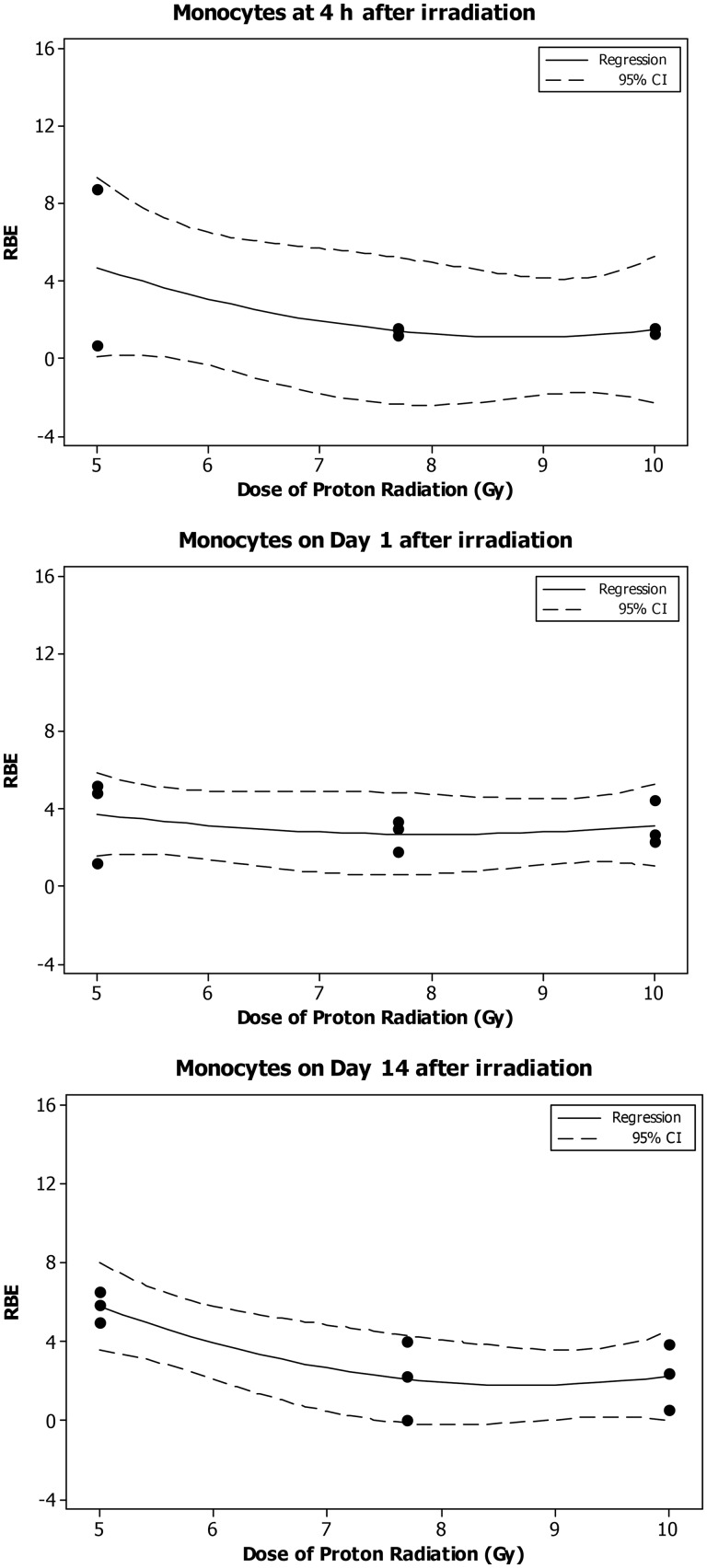
Trend of RBE values for the effects of SPE-like proton radiation on monocytes in Yucatan minipigs after irradiation. The fitted RBE values and associated 95% confidence intervals for the effect of simulated SPE radiation on monocytes are presented in Table 8. The curves are fitted using a quadratic model, and the 95% confidence interval is indicated by the dotted lines.

The monocyte count decreased significantly in a dose-dependent manner at 4 h and on Day 1 and 14 after the eSPE irradiation (Fig. 7). The linear component of the dose–response curve slope for 4 h and Day 1 and 14 after irradiation, was ∼ 63, 95 and <1% of the linear component of the proton dose–response curve slope for the corresponding time-points reported previously [14]. The steepest slope was observed at 4 h after irradiation. The quadratic component of the dose–response curve slope was negligible (≤1.930 × 10^−^^4^) for all time-points investigated except for Day 14. Based on the dose–response curve established in the animals irradiated with eSPE radiation (Fig. 7) and the monocyte count data reported previously for animals exposed to pSPE radiation [14], RBE values were calculated for the effect of pSPE radiation on monocytes. The RBE values exhibited a downward trend with the increase in the proton radiation dose at 4 h (Fig. 8A) and on Day 14 (Fig. 8C) after irradiation, whereas the trend on Day 1 (Fig. 8B) after irradiation was relatively flat. The fitted RBE values were > 1 for all three dose levels at all time-points after irradiation, although the lower limits of the 95% confidence interval for the RBE were only > 1 for 5 and 10 Gy on Day 1 (Fig. 8B) and 5 Gy on Day 14 (Fig. 8C) after irradiation. The RBE trend for monocytes was not evaluated for Day 4 and Day 30 after irradiation because no significant dose response was observed at these time-points after exposure to pSPE radiation [14] or eSPE radiation (Fig. 7).

**Table 7.** Relationship between RBE and proton radiation dose for neutrophils

Time after irradiation	Dose of proton radiation (Gy)	RBE		
		Fitted value	95% confidence interval	
			Lower limit	Upper limit
1 d	5	2.272	0.672	3.872
	7.7	2.564	0.964	4.164
	10	1.718	0.117	3.318
4 d	5	**5.025**	**3.644**	**6.407**
	7.7	2.230	0.849	3.612
	10	2.176	0.795	3.558
14 d	5	**5.980**	**4.541**	**7.419**
	7.7	**4.491**	**3.052**	**5.930**
	10	**2.609**	**1.171**	**4.048**

RBE was not calculated for 4 h or Day 30 after irradiation due to a lack of dose response in 6 + 12 MeV electron irradiated animals at these time-points.

**Table 8.** Monocyte counts in animals irradiated with 6 + 12 MeV electrons

Time after irradiation	Monocyte counts (fraction of control) in pigs irradiated at dose shown below											
	5 Gy		7.5 Gy		7.7 Gy		10 Gy		15 Gy		20 Gy	
	Mean ± SE	Change (%)	Mean ± SE	Change (%)	Mean ± SE	Change (%)	Mean ± SE	Change (%)	Mean ± SE	Change (%)	Mean ± SE	Change (%)
Pre-irradiation	1.00 ± 0.08	N/A	1.00 ± 0.08	N/A	1.00 ± 0.08	N/A	1.00 ± 0.08	N/A	1.00 ± 0.08	N/A	1.00 ± 0.08	N/A
4 h	0.49 ± 0.09	−50.6	0.45 ± 0.06	−55.1	0.55 ± 0.10	−45.4	0.34 ± 0.09	−66.1*	0.35 ± 0.01	−64.7#	0.26 ± 0.07	−73.6*
Day 1	0.67 ± 0.15	−32.6	0.44 ± 0.13	−55.9	0.54 ± 0.13	−46.2	0.42 ± 0.08	−54.0#	0.48 ± 0.15	−52.3	0.29 ± 0.08	−71.4*
Day 7	1.03 ± 0.06	3.2	0.90 ± 0.19	−10.0	2.47 ± 0.84	147.2***	0.62 ± 0.02	−18.5	1.27 ± 0.32	27.1	0.61 ± 0.06	−38.7
Day 14	0.98 ± 0.19	−2.3	1.12 ± 0.14	12.0	0.84 ± 0.23	−15.8	0.77 ± 0.20	−10.6	0.75 ± 0.09	−25.4	0.56 ± 0.16	−43.9
Day 30	0.44 ± 0.05	−56.3	1.62 ± 0.68	62.1	0.72 ± 0.16	−28.1	0.72 ± 0.18	−0.6	0.50 ± 0.04	−49.7	0.75 ± 0.16	−24.9

Statistical significance for comparison with the pre-irradiation value by the Tukey test is indicated by symbols of #(*P* < 0.10), *(*P* < 0.05), **(*P* < 0.01) and ***(*P* < 0.001), respectively.

**Fig. 9.**
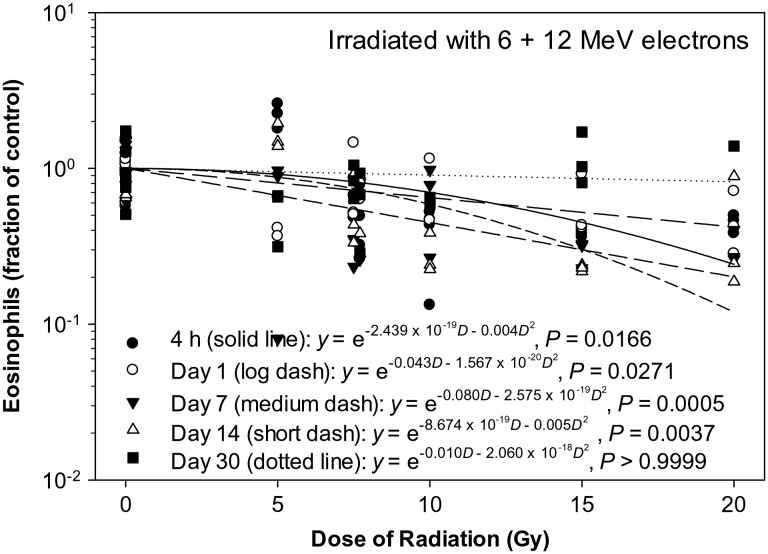
Dose–response relationship for eosinophils in Yucatan minipigs after electron irradiation. The eosinophil data for different electron radiation dose groups at different time-points after irradiation are presented in Table 9. The dose–response curve for each time-point after irradiation is fitted using a linear quadratic model.

**Fig. 10.**
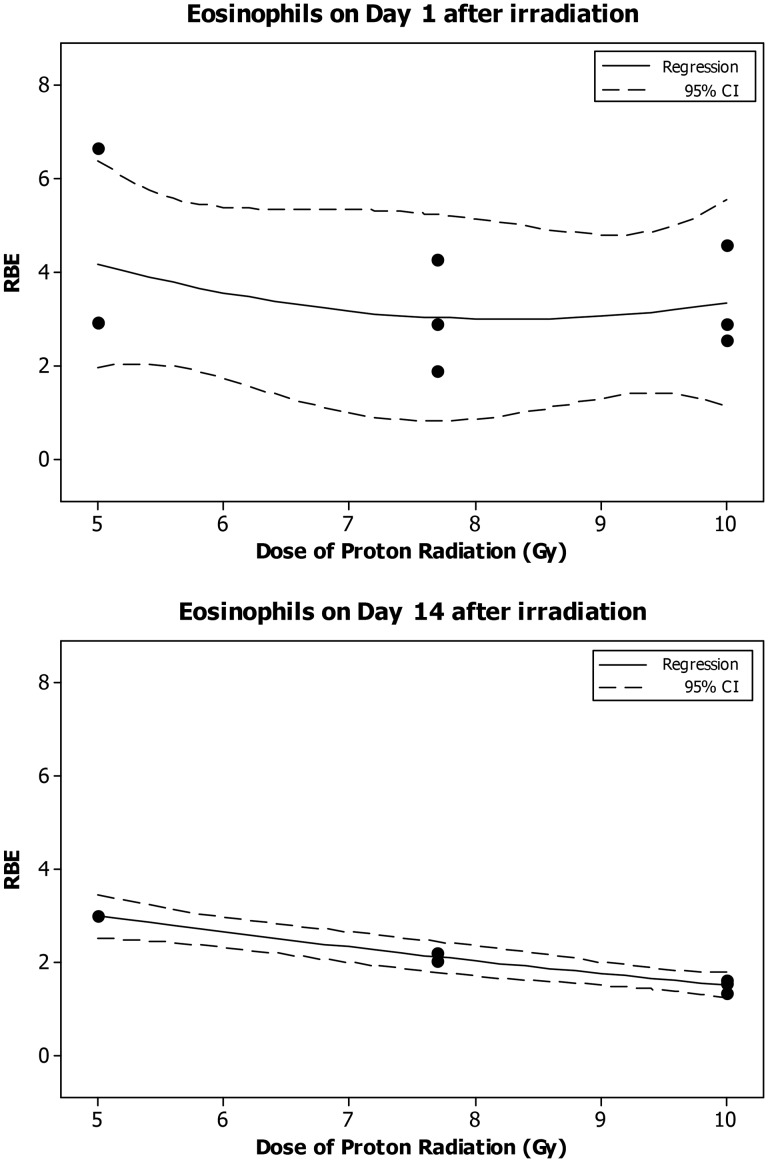
Trend of RBE values for the effects of SPE-like proton radiation on eosinophils in Yucatan minipigs after irradiation. The fitted RBE values and associated 95% confidence intervals for the effect of simulated SPE radiation on eosinophils are presented in Table 10. The curves are fitted using a quadratic model, and the 95% confidence interval is indicated by the dotted lines.

The eosinophil count decreased significantly in a dose-dependent manner at 4 h and on Day 1, 7 and 14 after the eSPE irradiation (Fig. 9). The linear components of the electron dose–response curve slope were <1, 30, 89 and <1% of the linear component of the proton dose–response curve slope for the corresponding time-points (4 h, Day 1, 7 and 14 after irradiation) reported previously [14]. The steepest initial slope was observed on Day 4 after irradiation; however, the downward slope accelerated faster at 4 h and on Day 14 due to the relatively large quadratic component of the dose–response curve slope at these time-points. The quadratic component of the dose–response curve slope was negligible (≤2.060 × 10^−^^18^) for all other time-points investigated. Based on the dose–response curve established in the animals irradiated with eSPE radiation (Fig. 9), and eosinophil count data reported previously for animals exposed to pSPE radiation [14], RBE values for the effect of pSPE radiation were calculated for eosinophils. The RBE trend for eosinophils was relatively flat on Day 1 (Fig. 10A) and downward on Day 14 (Fig. 10C) after irradiation. The lower limits of the 95% confidence interval for RBE values were > 1.00 for 5 and 10 Gy on Day 1 and for all three radiation dose levels on Day 14 after irradiation, indicating the RBE for the effect of pSPE radiation on eosinophils was significantly > 1.00 for these combinations of radiation doses and time-points. The RBE trend for eosinophils was not evaluated for 4 h or Day 4 and Day 30 after irradiation because no significant dose response was observed at 4 h or on Day 4 after exposure to pSPE radiation [14] or on Day 30 after eSPE irradiation (Fig. 9).

**Table 9.** Relationship between RBE and proton radiation dose for monocytes

Time after irradiation	Dose of proton radiation (Gy)	RBE		
		Fitted value	95% confidence interval	
			Lower limit	Upper limit
4 h	5	4.701	0.064	9.338
	7.7	1.417	−2.369	5.204
	10	1.456	−2.330	5.242
1 d	5	**3.723**	**1.598**	**5.848**
	7.7	2.694	0.569	4.819
	10	**3.147**	**1.022**	**5.272**
4 d	5	**11.11**	**5.96**	**16.27**
	7.7	5.08	−0.07	10.24
	10	3.43	−1.72	8.58
14 d	5	**5.787**	**3.561**	**8.014**
	7.7	2.085	−0.141	4.312
	10	2.247	0.021	4.474
30 d	5	2.01	−2.55	6.57
	7.7	1.99	−2.57	6.35
	10	1.56	−3.00	6.12

**Table 10.** Eosinophil counts in animals irradiated with 6 + 12 MeV electrons

Time after irradiation	Eosinophil counts (fraction of control) in pigs irradiated at dose shown below											
	5 Gy		7.5 Gy		7.7 Gy		10 Gy		15 Gy		20 Gy	
	Mean ± SE	Change (%)	Mean ± SE	Change (%)	Mean ± SE	Change (%)	Mean ± SE	Change (%)	Mean ± SE	Change (%)	Mean ± SE	Change (%)
Pre-irradiation	1.00 ± 0.13	N/A	1.00 ± 0.13	N/A	1.00 ± 0.13	N/A	1.00 ± 0.13	N/A	1.00 ± 0.13	N/A	1.00 ± 0.13	N/A
4 h	2.21 ± 0.23	121.4*	0.67 ± 0.11	−32.7	0.53 ± 0.13	−46.9	0.36 ± 0.12	−63.6	0.38 ± 0.01	−61.8	0.45 ± 0.03	−55.2
Day 1	0.48 ± 0.09	−52.0	0.83 ± 0.32	−17.1	0.60 ± 0.18	−40.3	0.70 ± 0.23	−30.5	0.59 ± 0.17	−41.0	0.47 ± 0.13	−55.6
Day 7	0.65 ± 0.28	−35.2	0.31 ± 0.04	−68.9	0.26 ± 0.13	−74.0	0.68 ± 0.21	−32.3	0.29 ± 0.03	−70.8	0.31 ± 0.05	−68.9
Day 14	1.61 ± 0.17	60.6	0.56 ± 0.18	−43.9	0.70 ± 0.16	−29.7	0.28 ± 0.05	−71.6	0.23 ± 0.01	−77.1	0.44 ± 0.23	−55.9
Day 30	0.55 ± 0.12	−45.4	0.84 ± 0.12	−16.0	0.63 ± 0.19	−36.8	0.61 ± 0.02	−38.6	1.18 ± 0.27	18.2	1.39 ± 0.13	39.2

Statistical significance for comparison with the pre-irradiation value by the Tukey test is indicated by symbols of #(*P* < 0.10), *(*P* < 0.05), **(*P* < 0.01) and ***(*P* < 0.001), respectively.

**Fig. 11.**
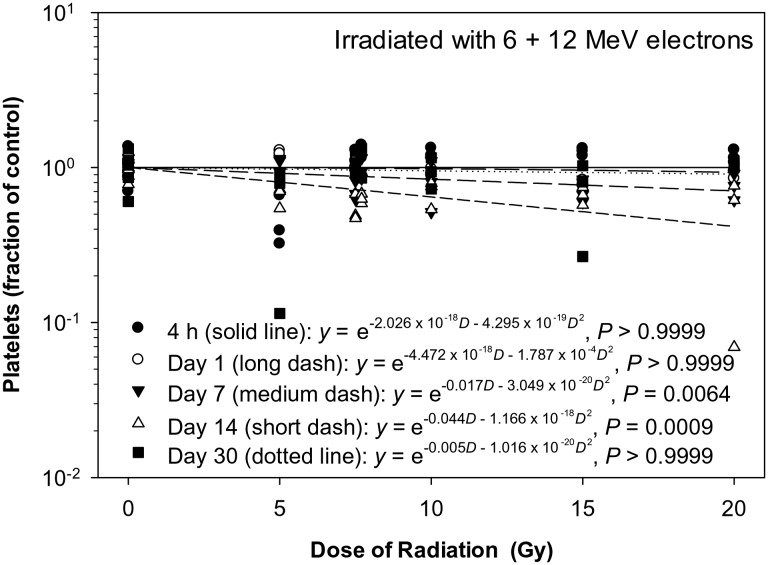
Dose–response relationship for platelets in Yucatan minipigs after electron irradiation. The platelet data for different electron radiation dose groups at different time-points after irradiation are presented in Table 11. The dose–response curve for each time-point after irradiation is fitted using a linear quadratic model.

**Fig. 12.**
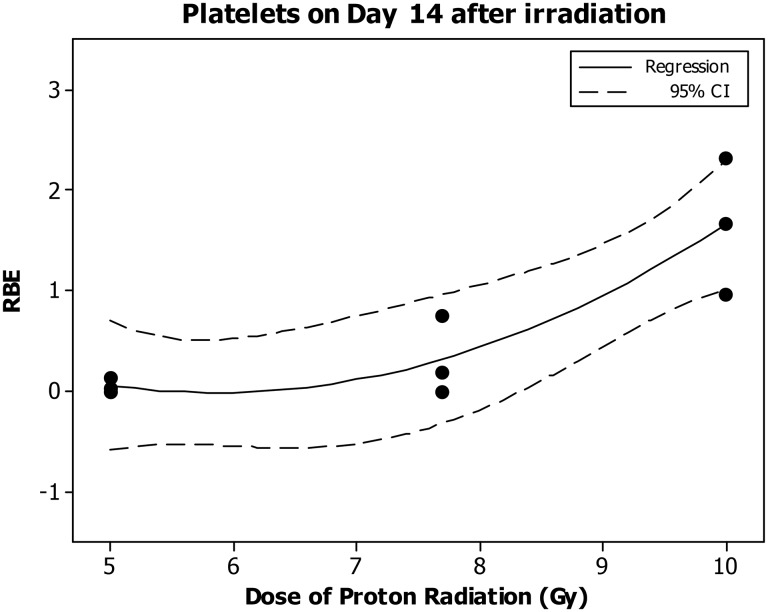
Trend of RBE values for the effects of SPE-like proton radiation on platelets in Yucatan minipigs after irradiation. The fitted RBE values and associated 95% confidence intervals for the effect of simulated SPE radiation on platelets are presented in Table 12. The curve is fitted using a quadratic model, and the 95% confidence interval is indicated by the dotted lines.

The platelet count decreased significantly in a dose-dependent manner only on Day 7 and 14 after the eSPE irradiation (Fig. 11A). The linear component of the dose–response curve slope was 0.017 and 0.044, respectively, for Day 7 and 14 with the steepest slope observed on Day 14 after irradiation. The quadratic component of the dose–response curve slope was negligible (≤ 1.787 × 10^−^^4^) for all time-points investigated. Based on the dose–response curve established in the animals irradiated with eSPE radiation (Fig. 11) and platelet count data reported previously for animals exposed to pSPE radiation [14], RBE values for the effect of pSPE radiation were calculated for platelets on Day 14 after irradiation. The fitted values for RBEs increased from 0.05 at 5 Gy to 1.65 at 10 Gy on Day 14 after irradiation (Fig. 12). The upper limits of the 95% confidence interval for the RBE were < 1.00 for 5 and 7.7 Gy whereas the lower limit of the 95% confidence interval was > 1.00 for 10 Gy, indicating that the RBE was significantly < 1.00 for 5 and 7.7 Gy whereas significantly > 1.00 for 10 Gy on Day 14 after irradiation. The RBE trend for platelets was not evaluated for other time-points after irradiation because no significant dose response was observed in animals exposed to pSPE radiation [14] and/or eSPE radiation at those time-points.

**Table 11.** Relationship between RBE and proton radiation dose for eosinophils

Time after irradiation	Dose of proton radiation (Gy)	RBE		
		Fitted value	95% confidence interval	
			Lower limit	Upper limit
4 h	5	**3.391**	**1.912**	**4.870**
	7.7	1.150	−0.058	2.358
	10	0.898	−0.310	2.106
1 d	5	**4.178**	**1.974**	**6.382**
	7.7	3.030	0.827	5.234
	10	**3.339**	**1.135**	**5.542**
14 d	5	**2.9837**	**2.5169**	**3.4505**
	7.7	**2.1191**	**1.7890**	**2.4491**
	10	**1.5112**	**1.2417**	**1.7807**

RBE was not calculated for Day 4 and Day 30 after irradiation due to a lack of dose response in 6 + 12 MeV electron irradiated animals at these time-points.

**Table 12.** Platelet counts in animals irradiated with 6 + 12 MeV electrons

Time after irradiation	Platelet counts (fraction of control) in pigs irradiated at dose shown below											
	5 Gy		7.5 Gy		7.7 Gy		10 Gy		15 Gy		20 Gy	
	Mean ± SE	Change (%)	Mean ± SE	Change (%)	Mean ± SE	Change (%)	Mean ± SE	Change (%)	Mean ± SE	Change (%)	Mean ± SE	Change (%)
Pre-irradiation	1.00 ± 0.05	N/A	1.00 ± 0.05	N/A	1.00 ± 0.05	N/A	1.00 ± 0.05	N/A	1.00 ± 0.05	N/A	1.00 ± 0.05	N/A
4 h	0.46 ± 0.23	−54.2**	1.10 ± 0.10	9.9	1.34 ± 0.03	34.4#	1.14 ± 0.13	14.1	1.27 ± 0.04	26.6	1.13 ± 0.10	13.4
Day 1	1.23 ± 0.09	23.1	1.05 ± 0.05	5.4	1.22 ± 0.03	21.9	1.02 ± 0.07	2.1	0.79 ± 0.02	−21.1	0.96 ± 0.07	−4.1
Day 7	1.06 ± 0.28	5.8	0.71 ± 0.06	−29.3	0.98 ± 0.09	−2.1	0.81 ± 0.17	−19.1	0.70 ± 0.07	−29.9	0.76 ± 0.09	−23.6
Day 14	0.66 ± 0.17	−34.4	0.55 ± 0.06	−45.5*	0.63 ± 0.03	−37.0#	0.69 ± 0.08	−30.6	0.66 ± 0.05	−33.9	0.48 ± 0.21	−51.8
Day 30	0.61 ± 0.12	−39.4	1.09 ± 0.10	9.1	1.15 ± 0.15	15.4	0.94 ± 0.12	−5.9	0.70 ± 0.23	−29.9	1.05 ± 0.03	4.9

Statistical significance for comparison with the pre-irradiation value by the Tukey test is indicated by symbols of #(*P* < 0.10), *(*P* < 0.05), **(*P* < 0.01) and ***(*P* < 0.001), respectively.

The RBC count did not decrease significantly at any of the time-points evaluated after the pigs were exposed to either eSPE radiation (data not shown) or pSPE radiation [14], and RBE values could not be calculated for RBCs.

## DISCUSSION

In a previous study, changes in the WBC, lymphocyte, neutrophil, monocyte, eosinophil, RBC and platelet counts in response to simulated pSPE radiation exposure at doses of 5, 7.7 and 10 Gy were evaluated in Yucatan minipigs [14]. In the animals exposed to pSPE radiation, WBCs, lymphocytes, neutrophils, monocytes and eosinophils decreased substantially within 24 h after irradiation in at least the two highest radiation dose groups (7.7 and 10 Gy), whereas platelets and RBCs did not decrease significantly in any radiation dose groups at any time-points after irradiation. In the present study, Yucatan minipigs were irradiated with eSPEs, which were used as a reference radiation to determine RBE values for the pSPE radiation. The RBE values calculated, based on the results obtained in the present study from the animals irradiated with eSPE radiation and the results obtained in the previous study from the animals exposed to pSPE radiation [14], indicate that RBE values for the pSPE radiation generally have a downward trend with increased dose for all blood cell types evaluated except RBCs, which were not significantly affected by exposure to either eSPE radiation or pSPE radiation, and platelets, which displayed an upward RBE trend at a single time-point (Day 14) after irradiation. The fitted RBE values calculated for WBCs, lymphocytes, neutrophils, monocytes and eosinophils were > 1.0 in all three radiation dose groups at all time-points evaluated, and the lower limits of 95% confidence intervals were > 1.0 in the majority of the radiation dose groups at different time-points. The slopes of the proton dose–response curves reported previously [14] were steeper compared with the slopes of the electron dose–response curves observed in the present study. Taken together, these results suggest that pSPE radiation is more effective than eSPE radiation at reducing the number of peripheral WBCs, lymphocytes, neutrophils, monocytes and eosinophils, especially at the low end of the 5–10 Gy dose range evaluated.

In the previous study with pSPE radiation at doses up to 10 Gy [14], the lowest leukocyte counts were observed at 4 h after irradiation for monocytes, on Day 1 for lymphocytes, on Day 4 for WBCs, between Day 4 and Day 14 for neutrophils, and between 4 h and Day 14 for eosinophils. Following the initial decrease after irradiation, WBC, lymphocyte, neutrophil, monocyte and eosinophil counts recovered slowly to levels that were not more than 38.3, 34.5, 47.8, 8.2 and 44.8% below the respective baseline control levels by Day 30 after irradiation. In the present study, the lowest leukocyte counts in the animals irradiated with eSPE radiation at doses up to 20 Gy occurred at 4 h after irradiation for monocytes, on Day 1 for lymphocytes, on Day 7 for WBCs, between Day 1 and Day 14 for neutrophils, and between 4 h and Day 14 for eosinophils, which were similar to the values observed in the animals exposed to pSPE radiation. By Day 30 after eSPE irradiation, WBC, lymphocyte, neutrophil, monocyte and eosinophil counts recovered to levels that were not more than 44.1%, 39.2%, 47.7%, 56.3% and 45.4% below the respective baseline control levels, which were also similar to the values observed in the animals exposed to pSPE radiation except for monocytes which recovered more quickly in the animals exposed to pSPE radiation than in the eSPE-irradiated animals. These results indicate that the time-courses for the changes in the leukocyte counts are comparable between the animals irradiated with eSPE radiation and the animals exposed to pSPE radiation; however, the radiation dose required to cause a similar extent of decrease in the leukocyte counts was higher for the eSPEs than it was for the pSPEs, as evidenced by the steeper slopes of the proton dose–response curves.

In the animals exposed to pSPE radiation, WBCs, lymphocytes, neutrophils, monocytes and eosinophils at the lowest level of the leukocyte counts decreased by 59.1, 89.5, 60.4, 73.2 and 75.5%, respectively, from the baseline control values obtained prior to the radiation exposure [14]. Reductions in peripheral leukocyte numbers contribute to a suppressed immune response, which can affect a mission or patient treatment in a clinical setting [15]. At the lowest level of the leukocyte counts in the animals irradiated with eSPE radiation at a dose of 10 Gy, which was the highest radiation dose used in the previous study with pSPE radiation [14], WBCs, lymphocytes, neutrophils, monocytes and eosinophils decreased by 56.2, 60.0, 54.0, 66.1 and 71.6%, respectively, which were ∼ 3–30% lower than the magnitudes of decrease observed for the respective leukocyte types in the animals exposed to pSPE radiation. These results agree with the findings supported by the > 1.0 RBE values and indicate again that pSPE radiation is more effective than eSPE radiation at reducing the numbers of peripheral WBCs, lymphocytes, neutrophils, monocytes and eosinophils. An average RBE was calculated based on all the available endpoints, resulting in an average RBE of 2.79 ± 0.23 (SEM), which is different from an RBE of 1.0, in a statistically significant manner. It is considered important that the RBE value is in fact different from 1.0 in a large mammalian model like that used in this report, since there are limited reports in which RBE values have been determined in biological models other than rodents or *in vitro* systems.

Based on the magnitude of the decrease and the time required to reach the lowest blood cell counts after irradiation in the present study, lymphocytes appeared to be the most sensitive cell type to the effects of the radiation exposure, which was also the conclusion reached previously in the study with pSPE radiation [14]. Also similar to the previous findings in the study with pSPE radiation was that the dose–response curves were generally linear on a logarithmic scale, and the quadratic components of the dose–response curve slopes were negligible except for eosinophils at a few selected time-points, indicating both the linear quadratic model and the multitarget model are suitable in most situations for accurate description of the dose–response relationship for the blood cell counts in irradiated Yucatan minipigs.

Due to technical limitations, the depth–dose beam profiles for the eSPE and pSPE could not be perfectly matched. Previous studies for ionizing-radiation-induced hematopoietic cell loss have used homogeneous dose delivery to the hematopoietic compartments (i.e. marrow, circulating blood/lymphatic volume, etc.). During an SPE, an inhomogeneous irradiation is delivered, which makes the interpretation of the results more complicated than in homogenous-dose-delivery experiments. We have developed a Geant4-based [16] Monte Carlo dose-modeling software tool that allows us to provide more accurate estimates for the dose to specific organs. A description of the dose calculation software will appear in a future publication and has been omitted here because it is beyond the scope of this work. Estimates of the median dose, mean dose and percentage of organ volume receiving 2 Gy for an animal with a nominal 5 Gy skin dose are shown in Table 15, including organs of skin, body (representing integral body dose), total lymphovascular volume (TLV, total body without skin) and bone marrow (marrow cavities of skull, spine, ribs, pelvis and proximal femurs and humeri). This analysis demonstrates that the dose distribution to the different organs are highly similar with a median difference of electron or proton dose across all volumes of 5.6%, which is very close to the allowable variation for human patients in prescribed vs actual dose (typically a 5% difference). Therefore, it is believed that the 5.6% variability is highly unlikely to contribute to any potential differences in hematopoietic responses that might be observed between eSPE and pSPE exposure in animals[Table RRT108TB13][Table RRT108TB14].

**Table 13. RRT108TB13:** Relationship between RBE and proton radiation dose for platelets

Time after irradiation	Dose of proton radiation (Gy)	RBE		
		Fitted value	95% confidence interval	
			Lower limit	Upper limit
14 d	5	**0.054**	**− 0.589**	**0.696**
	7.7	**0.315**	**− 0.328**	**0.958**
	10	**1.654**	**1.012**	**2.297**

RBE was only calculated for Day 14 after irradiation due to a lack of dose response in 6 + 12 MeV electron and/or 155 MeV proton irradiated animals at other time-points.

**Table 14. RRT108TB14:** RBC counts in animals irradiated with 6 + 12 MeV electrons

Time after irradiation	RBC counts (fraction of control) in pigs irradiated at dose shown below											
	5 Gy		7.5 Gy		7.7 Gy		10 Gy		15 Gy		20 Gy	
	Mean ± SE	Change (%)	Mean ± SE	Change (%)	Mean ± SE	Change (%)	Mean ± SE	Change (%)	Mean ± SE	Change (%)	Mean ± SE	Change (%)
Pre-irradiation	1.00 ± 0.02	N/A	1.00 ± 0.02	N/A	1.00 ± 0.02	N/A	1.00 ± 0.02	N/A	1.00 ± 0.02	N/A	1.00 ± 0.02	N/A
4 h	1.22 ± 0.04	22.1*	1.15 ± 0.02	15.3	0.93 ± 0.02	−7.0	1.06 ± 0.01	5.7	1.17 ± 0.01	16.5	1.13 ± 0.07	13.0
Day 1	1.10 ± 0.06	9.8	1.37 ± 0.02	37.1***	0.92 ± 0.03	−7.6	1.26 ± 0.08	26.4**	1.11 ± 0.05	11.1	1.14 ± 0.06	14.2
Day 7	1.08 ± 0.03	8.3	1.36 ± 0.01	36.0***	0.92 ± 0.04	−8.0	1.28 ± 0.05	27.5**	0.98 ± 0.02	−2.1	1.21 ± 0.03	20.5
Day 14	1.14 ± 0.05	13.5	1.21 ± 0.04	20.5*	0.90 ± 0.05	−10.1	1.16 ± 0.06	16.1	1.01 ± 0.00	1.0	1.14 ± 0.02	14.2
Day 30	0.75 ± 0.12	−25.4**	1.10 ± 0.02	10.4	0.90 ± 0.02	−9.8	1.11 ± 0.06	11.4	1.04 ± 0.04	3.6	1.19 ± 0.03	18.8

Statistical significance for comparison with the pre-irradiation value by the Tukey test is indicated by symbols of #(*P* < 0.10), *(*P* < 0.05), **(*P* < 0.01) and ***(*P* < 0.001), respectively.

**Table 15.** Monte Carlo-derived estimated doses to the integral body, skin, total lymphovascular volume (TLV) and bone marrow (BM) in an animal receiving 5 Gy skin dose

	Median Dose (Gy)		Mean Dose (Gy)		Organ Volume 2 Gy (%)	
	pSPE	eSPE	pSPE	eSPE	pSPE	eSPE
BODY	1.01	0.99	1.95	1.67	37.3	33.5
SKIN	4.81	4.41	4.74	4.32	95.4	96.9
TLV	0.85	0.85	1.60	1.41	30.1	27.2
BM	0.42	0.52	0.80	0.82	11.8	10.2

The percentage of the organ volume receiving 2 Gy is shown in the two far right columns.

Due to increased interest in proton radiotherapy for cancer treatment, a number of studies have attempted to define RBEs for protons using either the plateau or spread-out Bragg peak portions of the depth–dose distribution to create a relatively homogenous radiation dose for specific organ systems [17–19]. While the RBE values established in such studies for monoenergetic protons with homogeneous dose distribution are very useful for predicting the effectiveness of the proton radiotherapy relative to the effectiveness of photon radiotherapy, they are not necessarily accurate estimates of RBE values for protons in pSPE radiation, since RBE determinations for eSPE or pSPE radiation presents different challenges. Due to the inhomogeneous dose distribution of SPE radiation, with most of the protons in SPE radiation having energies ≤ 50 MeV, SPE radiation is expected to give a high superficial absorbed dose to the skin and significantly lower absorbed doses to internal organs [5], whereas X-rays and γ-rays are expected to result in relatively homogeneous absorbed doses to the whole body. Therefore, there is no way to match absorbed dose both to the whole body and to specific organ systems between SPE radiation and conventional radiation. Consequently, it would be uncertain whether any observed differences in irradiated animals are caused by the differences in absorbed dose distribution or the radiobiological effectiveness between SPE radiation and conventional radiation. The use of megavoltage electrons, which match the dose distribution of SPE radiation [10], removed this uncertainty and any observed differences in animals irradiated with pSPEs and pSPEs can be attributed to the radiobiological effectiveness between the two types of radiation. The linear energy transfer (LET) of secondary electrons produced through Compton scattering by ^60^Co and eSPEs produced by a linear accelerator is very similar. Theoretical and preclinical studies have consistently demonstrated that the RBE of megavoltage electrons is ∼ 1.0. In addition, clinical experience in human radiotherapy has confirmed an RBE value of ∼ 1 for megavoltage electrons [20–22]. While there certainly are some caveats in the application of electrons as a ‘conventional’ reference radiation for our studies, attempts to use ^60^Co gamma rays as a reference radiation would not be interpretable given the drastic dose distribution differences of SPE-like radiation and ^60^Co radiation. Thus, using eSPE as a reference radiation allows determination of meaningful RBE values that will help to determine the impact of the microdosimetric differences introduced by different particle identity while the macroscopic dose distribution is kept consistent. These RBE values determined in the present study for pSPE radiation using eSPE radiation as the reference radiation represent the only practical solution for attempting to leverage the vast knowledge accumulated from experimental and clinical research with conventional radiation, which suggests an RBE of 1 for protons, for assessing the risk of SPE radiation.

## CONCLUSION

In conclusion, the results obtained in the irradiated Yucatan minipigs indicate that pSPE radiation is more effective than eSPE radiation in damaging peripheral leukocytes; however, other characteristics of the response of leukocytes to radiation exposure, such as the time-course, the most sensitive cell type (the lymphocyte population), and the shape of the dose–response curves (generally log-linear), are similar between pSPE radiation and eSPE radiation. These findings provide additional evidence that eSPE radiation is a suitable reference radiation for determination of RBEs for the SPE and pSPE radiation, and the RBE estimation is not complicated by other characteristics of the response to the radiation exposure.

## FUNDING

This work was supported by the Center of Acute Radiation Research (CARR) grant from the National Space Biomedical Research Institute (NSBRI) through NASA NCC 9-58 and NIH Training Grant 2T32CA00967.
